# Spectroelectrochemical Studies of CTAB Adsorbed on Gold Surfaces in Perchloric Acid

**DOI:** 10.1021/acs.langmuir.2c03226

**Published:** 2023-02-08

**Authors:** José M. Gisbert-González, Valentín Briega-Martos, Francisco J. Vidal-Iglesias, Ángel Cuesta, Juan M. Feliu, E. Herrero

**Affiliations:** † Instituto de Electroquímica, Universidad de Alicante, E-03080 Alicante, Spain; ‡ Department of Chemistry, School of Natural and Computing Sciences, University of Aberdeen, AB24 3UE Aberdeen, Scotland, U.K.; § Centre for Energy Transition, University of Aberdeen, AB24 3FX Aberdeen, Scotland, U.K.

## Abstract

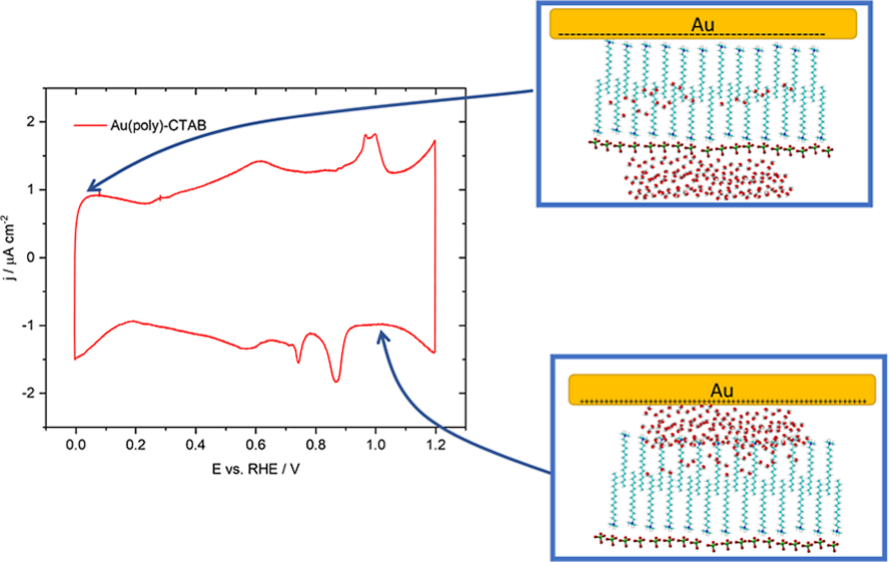

The behaviour of CTAB adsorbed on polycrystalline gold electrodes has been studied using a combination of spectroelectrochemical methods. The results indicate that the formation of the layer is the consequence of the precipitation of the CTAB micelles on the electrode surface as bromide ions, which stabilize the micelles, are replaced by perchlorate anions. This process leads to the formation of CTA^+^ layers in which perchlorate ions are intercalated, in which the adlayer suffers a continuous rearrangement that leads to the formation of micro-dominions of different types of hydrogen-bonded water populations throughout the adlayer. After prolonged cycling, a stable situation is reached. Under these conditions, water molecules permeate through the adlayer toward the electrode surface at potentials positive of the potential of zero charge, due to the repulsion between the CTA^+^ layer and the positive charge of the electrode.

## 1 Introduction

The adsorption of organic molecules on different metals has experienced an up-growing interest due to its wide number of potential applications in corrosion prevention,^1^ as molecular immobilizers to build sensors^2^ or for nanoparticle synthesis.^3^ In this context, cetyltrimethylammonium bromide (CTAB) is one of the most commonly used additives in the growth of metallic nanoparticles.^4−6^ The cetyltrimethylammonium cation (CTA^+^) consists of a positively charged head with three methyl groups bonded to a nitrogen atom and a hydrophobic hexadecyl tail, giving rise to surfactant properties. In fact, when the CTAB is dissolved beyond its critical micelle concentration (CMC), ∼10^–3^ M, CTA^+^ cations form surface-charged, spherical androd-shaped micelles that are partially ionized and surrounded and stabilized by a cloud of counterions (Br^–^). The ratio of the freely dissociated counterions in solution indicate the so-called degree of micellar counterion dissociation (α).^7−9^ Therefore, in this work, a CTAB micelle or adlayer conformation is referred to a configuration in which the cations and surrounding counterions are involved, and as CTA^+^ adlayer when only the cation is involved.

**Table 1:** IR Band Assignment

stretching modes	ν/cm^–1^	bending modes	ν/cm^–1^
*ν*(OH)	3750–3100	δ(HOH)^a^	1750–1530
ν_asym_(N–CH_3_)	3015	δ(CH_2_)-scissoring	1447
ν_asym_(CH_3_)	2958	δ_sym_(N^+^−CH_3_) + δ_sym_(N^+^–CH_3_)-umbrella	1379
ν_asym_(CH_2_)	2923	δ(CH_2_)-twisting	1297
ν_asym_(CH_2_) FR	2900		
ν_sym_(CH_2_)	2853		

a H_2_O and H_3_O^+^ bending modes included in the assignment.

**Table 2:** Raman Band Assignments

stretching modes	Raman shift/cm^–1^	bending modes	Raman shift/cm^–1^
ν_asym_(N–CH_3_)	3015	δ_sci_(CH_2_)scissoring	1494
ν_sym_(N–CH_3_)	2968	δ_sci_(CH_2_)+δ_def_(CH_3_)	1435
ν_asym_(CH_2_)	2925	δ_wag_(CH_2_)	1349
ν_asym_(CH_3_)	2862	δ_tw_(CH_2_)-twisting	1267
ν_asym_(CH_2_) FR	2895	δ_wag_(CH_2_)	1146
ν_sym_(CH_3_)	2850		
ν_sym_(CH_2_)	2843		
ν(C–C)	1078		
ν(Cl–O)	940		
ν(N–CH_3_)	768		

This property plus its tendency to bond strongly to gold surfaces has caused CTAB to be widely used for the synthesis of gold nanoparticles (AuNPs) and, more specifically, gold nanorods.^10^ CTAB was found to form bilayers on the gold surface during the synthesis process, with one monolayer being adsorbed through its polar head and interacting with the other one through their hydrophobic tails.^11,12^ The presence of counterions through the adsorption/synthesis processes plays a key role in the size and shape of AuNPs.^13−16^ Nevertheless, the conformation of the bilayer and how it is packed, and hence the final shape of the AuNPs, depends on multiple parameters like the concentration of the surfactant, the ionic strength of the solution, and the chemical nature of additional species present in it, among other factors.^17^ Yet, the Au-CTAB interphase in HClO_4_ is not well understood. Most studies have been aimed at biomedical applications, focusing on eliminating or replacing CTAB from the AuNPs surface, mainly because of its cytotoxic properties, a consequence of its capability to permeate through cellular membranes.^18,19^

Based on all this, the adsorption behaviour of CTA^+^ has been studied on gold single-crystal electrodes, particularly on Au(111), Au(100), and Au(110).^20,21^ CTAB adsorbs spontaneously on gold surfaces. Upon cycling, bromide desorbs leading to the formation of CTA^+^ in perchloric acid solutions.^20^ Furthermore, CTA^+^ experiences an attachment/detachment process modulated by the charge density on the electrode surface. As the head of CTA^+^ is positively charged, the molecule is electrostatically repelled from the surface at potentials more positive than the pzc, without being diluted into the bulk of the solution, and re-attaches back to the surface if the charge density is made negative again. The reversibility of this process and the subsequent stability of CTA^+^ adlayers is affected by the pH and the presence of anions embedded in the adlayer.^21^ When perchloric acid solutions are used, ClO_4_^–^ anions strongly bond to the surfactant head group breaking the micelles and forming a slightly soluble salt bonded to the gold surface.^22^ The detachment process causes water molecules to permeate through the layer towards the surface. This phenomenon is in agreement with previous studies with biomimetic phospholipid layers adsorbed on metal surfaces reported by Lipkowski.^23^ This long-term stability of CTA^+^ adlayers can be exploited in the development of biosensors for fast drug screening and selective detection of ions, among others.^24,25^ The behavior of the CTA^+^ layers differs from that observed for bromide salts of quaternary ammonium ions with shorter alkyl chains, where, in the absence of bromide, the ammonium ion desorbs from the gold surface.^26^

In this context, this work is focused on the study of the Au-CTAB and Au-CTA^+^ interactions by using spectroscopic techniques namely, infrared reflection-absorption spectroscopy, attenuated total reflectance-surface-enhanced infrared absorption spectroscopy (ATR-SEIRAS) and surface-enhanced Raman scattering (SERS) to gain insight into the stabilization and conformation of the adlayer.

## 2 Experimental Section

Electrochemical experiments were carried out in a glass cell with a reversible hydrogen electrode (RHE) as the reference electrode and a gold counter electrode. All potentials in the text are referred to the RHE scale. Supporting electrolyte solutions were prepared using concentrated perchloric acid (Merck Suprapur), and ultrapure water (18.2 MΩ cm, Elga Vivendi). CTAB solutions were prepared using CTAB (BioUltra, for molecular biology, ≥99.0%, Sigma-Aldrich). All solutions were deoxygenated with Ar (N50, Air Liquide) except for the ATR-SEIRAS experiments, where the purge was done using N_2_ (BOC, Research Grade N5.5). Voltammetric experiments were carried out at room temperature using a wave signal generator (EG&G PARC 175), potentiostat (eDAQ 161), and digital recorder (eDAQ e-corder 401) workstation. Cyclic voltammograms were recorded at 50 mV s^–1^. All the experiments were done at room temperature.

ATR absorbance experiments were performed using a Nexus 8700 (Thermo Scientific) spectrometer equipped with an MCT-A detector and a wire grid ZnSe polarizer (Pike Tech) using p-polarized light. A ZnSe prism bevelled at 45° was placed at the top of a Veemax (Pike Tech.) reflectance accessory. A resolution of 8 cm^–1^ and 50 interferograms was used to collect every spectrum.

ATR-SEIRA spectra were recorded using a Nicolet iS50R FTIR spectrometer equipped with a liquid nitrogen-cooled MCT detector and a homemade ATR accessory, using unpolarized light. The working electrode was a Au film deposited on the totally reflecting plane of a Si prism bevelled at 60° following a previously reported procedure.^27^ The Si prism was attached to the spectroelectrochemical cell using an O-ring seal and electrical contact with the film was made by pressing onto it a circular gold wire. Before any ATR-SEIRAS experiment, the film was immersed in a 10^–2^ M CTAB solution for 10 s, then, it was mounted in the cell and cycled in 0.1 M HClO_4_ for different times according to the respective experiment set-up conditions. The potential-step spectra were collected with a resolution of 4 cm^–1^ and 200 interferograms at each potential. Specific details of the potentiodynamic spectra are described in the Results and Discussion section when describing each experiment.

Differential spectra are calculated as −log , where *R*_reference_ and *R*_sample_ are the reference and sample spectra, respectively. Positive bands correspond to species present in the sample spectrum that were absent in the reference spectrum, while negative bands correspond to species present in the reference spectrum that are absent in the sample spectrum. For all the figures, the conditions for the reference spectrum are given.

SERS measurements were performed using the so-called nanoparticles-on-electrode approach.^28−30^ The Au nanostructured electrodes were made by depositing a droplet of the corresponding aqueous suspension of the metal nanostructure onto a polycrystalline polished Au disk (3 mm in diameter) sheathed in a threaded poly-(tetrafluoroethylene) (PTFE) piece by using a pipette. The droplet was dried in air and the substrate was then mounted on a PTFE flow cell specifically designed for the in situ Raman measurements. In electrochemical environments, a KCl-saturated Ag/AgCl electrode was used as a reference electrode and an Au wire was used as the counter electrode. Raman spectra were obtained with a NRS-5000 laser Raman spectrometer (Jasco). The excitation line used was a 17 mW He–Ne laser at 632.8 nm. The laser beams were focused through a ×50 LWD objective (0.5 NA) into a 2 μm spot at the electrode surface. The spectrometer resolution was better than 5 cm^–1^ and the detector was a Peltier cooled charge coupled device (1024 × 256 pixels).

## 3 Results and Discussions

### 3.1 ATR Absorbance Spectrum of CTAB

**Figure 1:**
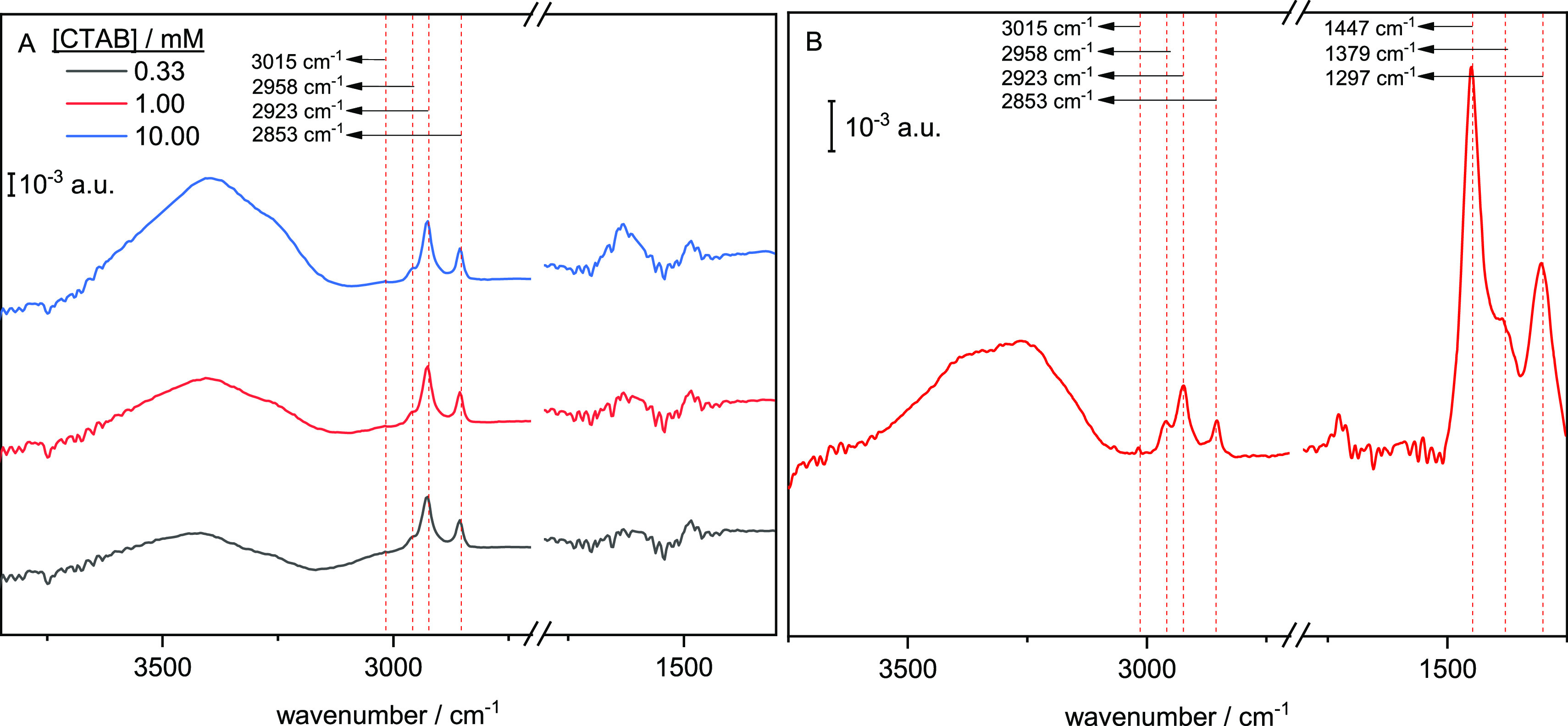
(A) ATR absorbance spectra of 3.3 × 10^–4^ M (black), 10^–3^ M (red) and 0.01 M (blue) aqueous CTAB solutions. (B)ATR absorbance spectrum of CTAB precipitated from a 10^–3^ M aqueous solution. Pure water was used as the reference spectrum in all cases. The vertical lines show the main bands in the alkane stretching region. 50 interferograms were taken for each spectrum.

Before explaining the spectral behaviour of CTA^+^ adsorbed on the surface, it is important to determine the main bands of CTAB in solution. For this reason, reference spectra of CTAB were taken. Figure 1A shows ATR absorbance spectra of dissolved CTAB at different concentrations. The assignment of the main absorption bands in the spectra is summarized in Table 1. The water stretching, ν(OH), and bending, δ(HOH), modes which appear in the regions at 3750–3100 cm^-1^ and 1700–1530 cm^–1^, respectively, grow with the concentration. On the other hand, the alkane stretching bands at 3000–2850 cm^–1^ are independent of the concentration. In this region, the main bands are the asymmetric CH_2_ stretching (ν_asym_(CH_2_)) at 2923 cm^–1^ and the symmetric CH_2_ stretching (ν_sym_(CH_2_)) at 2853 cm^–1^. This exact position indicates the formation of micellar clusters.^31^ The ν_asym_(CH_3_) appears as a shoulder at 2958 cm^–1^ and the ν_asym_(N–CH_3_) at 3015 cm^–1^ is also visible. The band centred at 1480 cm^–1^ is due to combination mode δ(CH_2_) scissoring + δ_asym_(N–CH_3_) and is also independent of the concentration. The independence of the intensities related to the CTA^+^ cation with the concentration of CTAB suggests that the CTAB micelles should be fused on the surface of the prism. It should be taken into account that infrared absorption arises from the species present within the penetration length of the evanescent wave. In the absence of a metal film deposited on the surface of the ATR element, this wavelength-dependent penetration varies roughly from ca. 150 nm around 4000 cm^–1^ to 700 nm around 1000 cm^–1^ so, even if micelles substitute a monolayer film, the evanescent wave will still be able to probe all of the CTA^+^ ions originally in the monolayer and now in the micelles. On the other hand, for the highest concentration, a large increase in the water signal is observed, which is consistent with the formation of micelles, as this will allow more water molecules to approach closer to the electrode surface. It should be stressed that this concentration is above he CMC.

Figure 1B shows an ATR absorbance spectrum of CTAB precipitated from a 1 mM solution. A certain volume that was added to the prism was cooled down below its saturation temperature (∼30 °C) originating the CTAB to precipitate. Here, the shape of ν(OH) water bands changes slightly from the previous one, thus, indicating that the state of the CTAB must modify the water structure in the surroundings. The δ(HOH) mode is centred at 1727 cm^–1^, which is blue-shifted from the usual frequency and cannot be considered only related to water bending modes. The ν(CH) bands are essentially the same as those described before except for the ν_asym_(CH_3_) (2958 cm^–1^), which is significantly sharper. The CH_2_ bending region is dominated by the band at 1447 cm^–1^ of the δ(CH_2_) scissor mode.^32^ A shoulder also appears at 1379 cm^–1^, which corresponds to the δ_sym_(N^+^–CH_3_) and the δ_sym_(N^+^–CH_3_) (umbrella).^32,33^ An additional band at 1297 cm^–1^, which is not well identified, could be related to the wagging-twisting progression series of the methylene chain.^34^ Clearly, the precipitation of CTAB shows significant differences in the CH_*x*_ bending region, related to the conformational changes in the CTAB structures.

### 3.2 Electrochemical Behaviour of Au(poly)-CTAB in Acidic Solution

**Figure 2:**
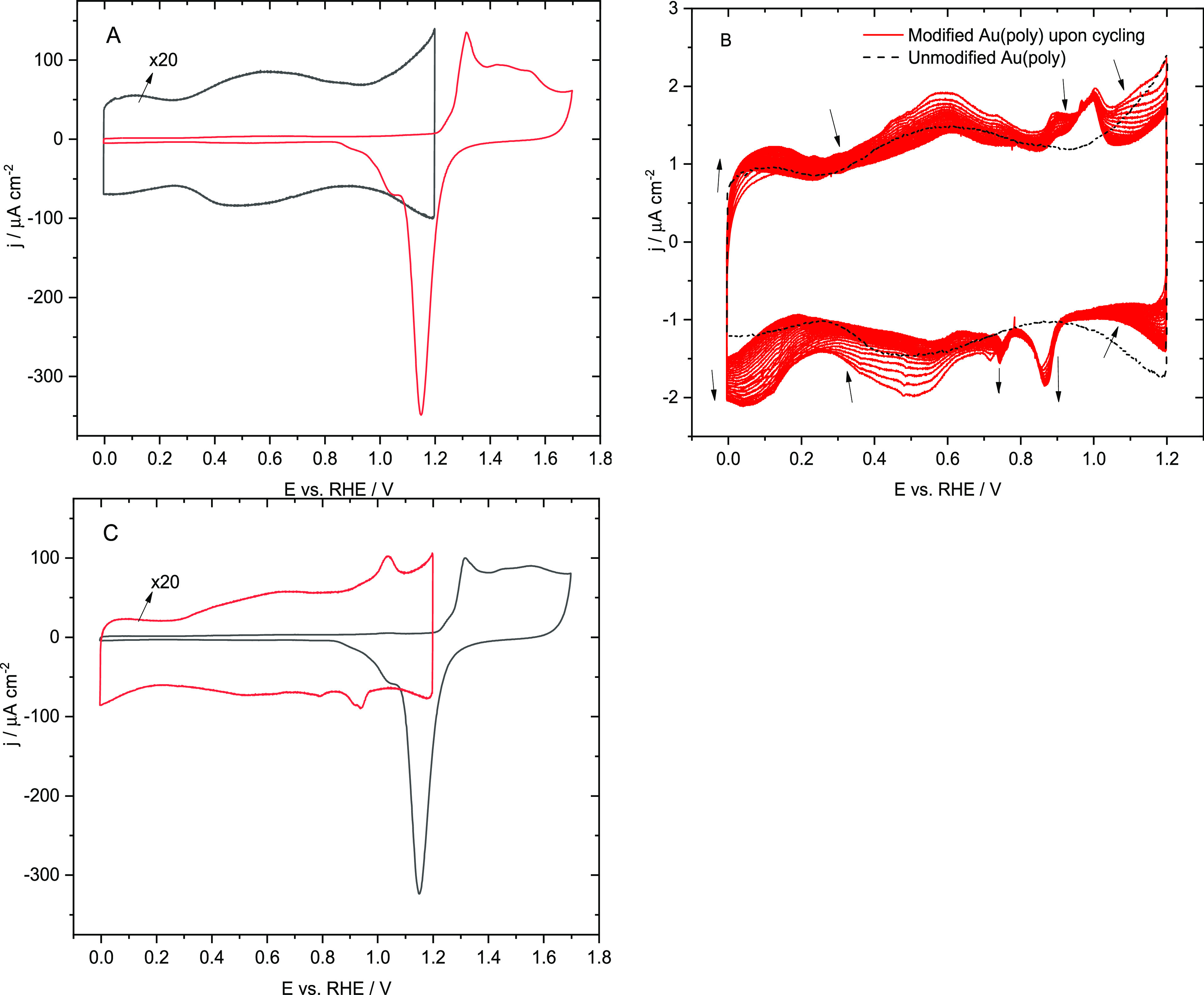
Voltammetric profiles of (A) unmodified polycrystalline Au, and (B,C) CTAB-modified polycrystalline Au in 0.1 M HClO_4_. The evolution upon continuous cycling with a positive potential limit of 1.2 V is illustrated in (B), while (C) shows the profile when the positive potential limit is increased to 1.7 V and the subsequent profile if the limit is set again to 1.2 V immediately after the excursion up to 1.7 V and back to 0 V. ν = 50 mV s^–1^.

Figure 2 shows the voltammetric profiles of an unmodified (Figure 2A) and a CTAB-modified polycrystalline gold electrode (Figure 2B,C) in 0.1 M HClO_4_. Modification with CTAB was achieved by immersing the electrode at open-circuit after flame-annealing in a 10^–3^ M CTAB solution for 10 s. For the unmodified electrode, keeping the positive potential limit below 1.2 V (Figure 2A, black line), results in a voltametric profile symmetrical with respect to the *x*-axis, characteristic of double-layer charging and indicating that no specific adsorption occurs. In the same potential region, the voltammetric profile of the CTAB-modified electrode (Figure 2B) changes continuously upon cycling. The double-layer charging current, which is initially higher than in the unmodified electrode in most of this potential region, decreases with cycling, while the peaks appearing between 0.85 and 1.0 V (positive scan) and between 0.78 and 0.93 V (negative scan) become sharper and better defined. After some cycling, a stable profile is reached, indicating that no significant desorption of CTA^+^ is occurring. This evolution can be assigned to the desorption of bromide anions initially present within the CTA^+^ adlayer and the concomitant exchange with ClO_4_^–^, causing a rearrangement of the adlayer. It should be noted that Br^–^ anions should desorb at low potentials on Au(111) electrodes (ca. at 0.1 V in this solution). The successive cycling of the electrode within this range in the absence of bromide in solution should lead to the progressive and complete removal of bromide ions from the interphase by diffusion to the bulk solution.^35^ Also relevant regarding the adlayer behaviour are the changes (or rather lack thereof) in the oxide formation/reduction region beyond 1.2 V. For the unmodified electrode (Figure 2A), the voltammetric profile shows a sharp peak at 1.32 V followed by a broad wave which have been assigned to the formation of an OH monolayer followed by the formation of a surface oxide.^36,37^ In the negative sweep, the surface gold oxide is reduced in a single peak. Except for a slight diminution in the intensity of the peak at 1.32 V and of the reduction peak, these features remain unaltered after modifying the electrode with CTAB (Figure 2C). Interestingly, the voltammetric profile obtained in the potential region below 1.2 V after oxidising the surface by extending the positive potential limit to 1.7 V is essentially identical to the stationary profile obtained by continuously cycling up to 1.2 V (Figure 2B). As demonstrated for single crystal electrodes,^20^ this behaviour can be explained by the formation of a stable CTA^+^ layer after expelling the Br^–^ anions. At sufficiently negative potentials, the negative charge on the electrode surface maintains the adlayer attached to it. As the potential increases and the surface charge becomes positive, the adlayer detaches and water permeates through it. If the oxidation region of Au is reached, the water layer in contact with the electrode surface gives rise to oxidation/reduction peaks that are essentially the same as those recorded in the absence of CTA^+^. Moreover, when the electrode surface charge becomes negative again the adlayer reattaches, without alterations.

### 3.3 Potentiostatic ATR-SEIRAS Experiments

**Figure 3:**
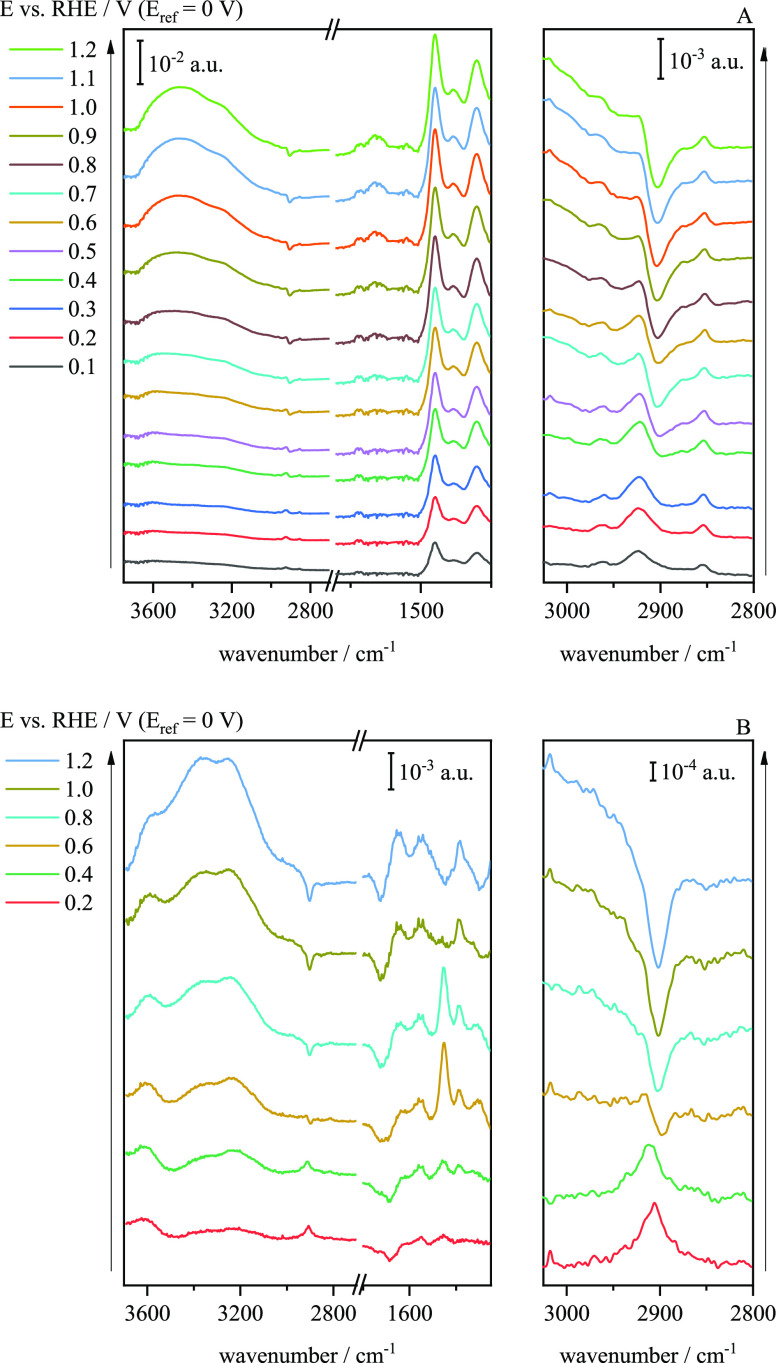
Potential-dependent ATR-SEIRA spectra (*E*_ref_ = 0 V) of CTAB adsorbed on a gold film chemically deposited on the Si prism in 0.1 M HClO_4_ before any voltametric cycle (A) and after a cyclic voltammogram between 0 and 1.7 V (B). The CH_x_ stretching region is plotted on the right. Reference spectra were taken at 0 V in each series of spectra.

In order to gain insight into the nature of the CTA^+^ adlayer, and its transformation upon cycling, IR experiments in different configurations were carried out. In the first set of experiments, the structure of the adlayer at different stages during its transformation is analysed using ATR-SEIRAS. Figure 3A shows ATR-SEIRA spectra of the CTAB adlayer immediately after adsorption on a chemically deposited gold film on Si at increasingly positive potentials within the region between 0.1 and 1.2 V. The reference spectrum was taken at *E*_ref_ = 0 V.

The first spectrum taken at 0.1 V shows relatively intense bands below 1500 cm^–1^ and in the water stretching region above 3000 cm^–1^ that must be the consequence of significant changes in the adlayer due to changing the potential from 0 to 0.1 V. These signals increase with increasingly positive potential. Examining in detail the region corresponding to the CH_2_ bending modes, it can be observed that the position of the bands and their relative intensity is very similar to that observed for precipitated CTAB (Figure 1B). The observed behaviour can therefore be assigned to the formation of a CTAB layer with the same characteristics as the precipitate. The solubility of CTA-perchlorate in aqueous solutions is low. It is therefore reasonable to propose that the formation of the precipitate-like adlayer is triggered by the substitution of the bromide anions surrounding the CTA^+^ cations, which stabilize the formation of micelles, by perchlorate. Beyond the pzc, the surface becomes positively charged so that perchlorate and bromide anions are attracted to it. Nevertheless, Br^–^ is electrostatically repelled from the surface and replaced by ClO_4_^–^ during the continuous cycles due to absence of bromide in the supporting electrolyte (which creates a concentration gradient that drives bromide away from the surface). This process can be accelerated at low potentials, where the charge of the surface is negative, and anions are repelled by the negative surface charge.^20^ Thus, it can be proposed that initially CTAB adsorbs forming micelles on the surface, which open in the presence of perchlorate to form a stable adlayer as bromide anions are progressively displaced by perchlorate. It has been shown by Brosseau et al.^38^ that quaternary ammonium ions form ion-pairs with their counter-ions on Au(111) near the *E*_pzc_, which agrees with our previous studies using the laser-induced temperature pulse method.^21^

Regarding the other spectral region where significant changes are observed, that of the water stretching modes, the main band is centred at 3450 cm^–1^ with a shoulder at ∼3250 cm^–1^. These bands are related to hydrogen-bonded multimer water and a network of hydrogen-bonded water, respectively.^39,42^ On the other hand, the region ascribed to less hydrogen-bonded water between 3600 and 3550 cm^–1^ also grows in intensity at *E* > 0.7 V, which is the main difference compared to the spectra of the precipitated CTAB (Figure 1B). When CTAB precipitates, the water inside the micelles is released into the bulk, leading to an increase in hydrogen-bonded water. On the other hand, the water inside the micelles that is released during the initial formation of the adlayer is less hydrogen-bonded than bulk water, probably because some water molecules are trapped within the adlayer or between the adlayer and the electrode.

Additional bands are also observed in Figure 3A in the CH_x_ stretching mode region. At the lowest potentials, the spectra are very similar to that observed for the precipitated CTAB but less defined. However, a negative band appears centred at 2900 cm^–1^ at *E* > 0.3 V, ascribed to the ν_asym_(CH_2_) mode and its Fermi resonance.^40^ As will be shown later, this band is associated with the detachment of the adlayer induced by the increasingly positive surface charge.

To demonstrate that the observed changes are related to the initial formation of the CTA^+^ layer, the electrode is cycled up to 1.7 V to accelerate the removal of bromide ions from the adlayer. Then, a new set of spectra is acquired (Figure 3B) between 0.1 and 1.2 V using a new reference spectrum taken at 0 V. As can be seen from the comparison between Figures 3A and 3B, significant differences can be observed. First, the bands for the bending modes of CH_x_ are significantly less prominent. Moreover, the relationship between the different modes differs from that obtained for the precipitated CTAB. The bands characteristic of water also show significant differences. Before analysing them, it must be highlighted that water bands will be interpreted based on the average number of hydrogen bonds of the water molecules involved in a given signal. Some of them will be mainly isolated whereas other will form water dimers to tetramers.^41^ These types of water populations are termed as water micro-dominions. Concerning this, for a specific absorption band, the lower the number of hydrogen-bonds a water molecule has, the higher its frequency. Thus, bands related to isolated water in Figure 3B are proportionally more intense than those on Figure 3A. On the other hand, the bands related to the stretching CH_*x*_ vibrating modes follow the same tendency as shown in Figure 3A. Finally, CH_*x*_ bending modes show the apparition of a sharp band related to the δ(CH_2_)-scissor mode in the 0.4 < *E* < 1.0 V potential region, implying a change in the adlayer conformational structure. 0.4 V is close to the pzc, indicating that the surface charge plays a key role in the adlayer behaviour. As will be shown later when discussing the spectra of the stabilized adlayer, the spectra in Figure 3B contain features common to both the initial film and the stabilized adlayer. Thus, it can be said that the process leading to the formation of the CTA^+^ layer is largely complete after the first scan due to the substitution of bromide by perchlorate anions, which leads to the disruption of the micellar structure of the CTAB film.

**Figure 4:**
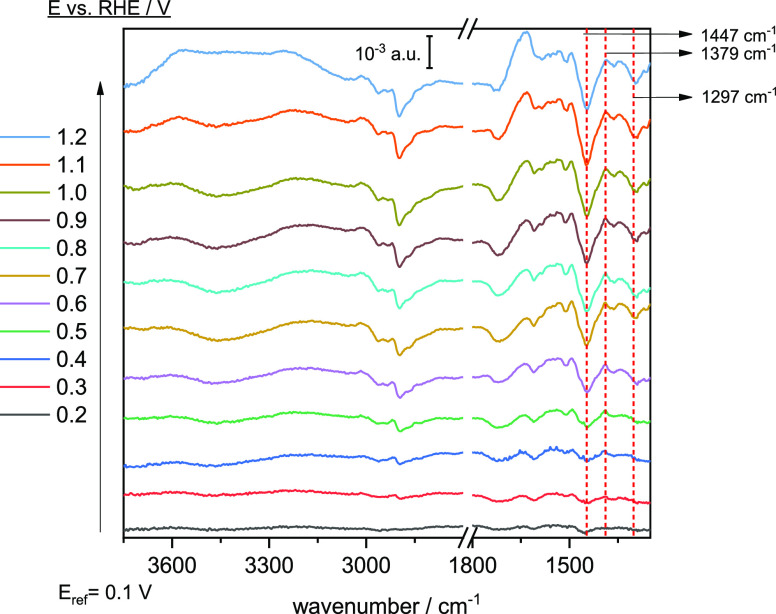
Potential-dependent ATR-SEIRA spectra in 0.1 M HClO_4_ of adsorbed CTAB adsorbed on a gold film chemically deposited on Si recorded after 100 cycles between 0 and 1.2 V at 20 mV s^–1^. The reference spectrum was recorded at 0.1 V.

To determine the evolution of the adlayer with potential once it reached a stable configuration, the modified electrode was cycled 100 times at 20 mV s^–1^ and a new series of spectra was recorded, using the spectrum at 0 V in this series as the reference spectrum (Figure 4). Negative bands are observed in the ν(C–H) region and their intensity increases with the potential. The shape of the bands is very similar to that measured for CTAB in solution. This behaviour was attributed to the potential-induced reversible detachment of the adlayer when the electrode charge becomes positive. In addition, negative bands are observed in the δ(CH_2_) region. Here, the δ(CH_2_)-scissor mode at 1447 cm^–1^ and the band associated with the twisting of CH_2_ at 1297 cm^–1^ are clearly negative. However, the δ_sym_(N^+^–CH_3_)-related band at about 1379 cm^–1^ is positive, which is due to a change in the conformation of the N^+^–(CH_3_)_3_ group so that the dynamic dipole moment of this mode becomes more perpendicular to the surface. Probably, the detachment of the N^+^–(CH_3_)_3_ group leads to a smaller angle between the surface normal and the N–C bonds. On the other hand, the intensity of the bands related to the vibrational modes of water increases with the potential, with the only exception of the band at 1720 cm^–1^, which is related to the hydronium ions. When the charge becomes positive, the hydronium ions are repelled from the surface, which explains the negative band. In the ν(OH) region, two positive bands are observed at ∼3600 and ∼3200 cm^–1^ at *E* < 0.5 V. As mentioned earlier, these bands are related to isolated water molecules and the hydrogen-bonded network water, respectively, indicating that two different water structures form as the potential increases. The hydrogen-bonded network water is likely formed in contact with the electrode and the isolated water is likely interstitial water in the adlayer. Above 0.8 V, positive bands appear at about 3450 cm^–1^, suggesting that a different, less hydrogen-bonded water structure forms when the CTA^+^ layer is detached. This type of water should form when the CTA^+^ adlayer is being detached from the surface. It is ejected from the adlayer when it precipitates into a CTAClO_4_ film during detachment.

### 3.4 Potentiodynamic ATR-SEIRAS Experiments

**Figure 5:**
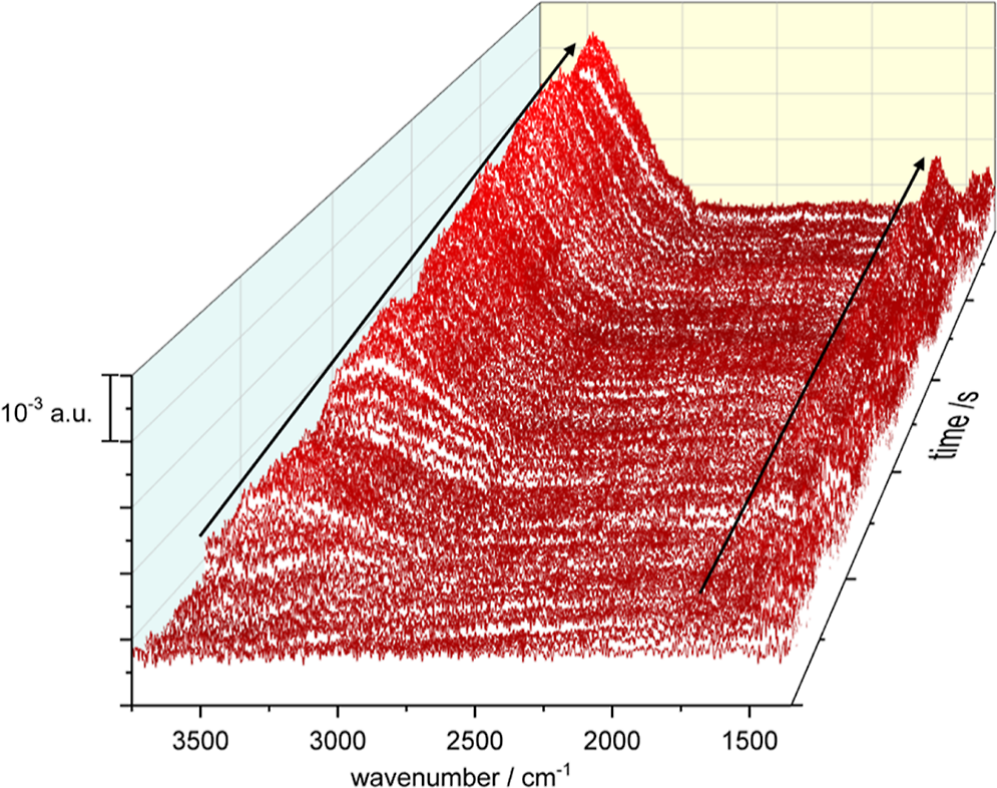
ATR-SEIRA spectra of CTAB on Au in 0.1 M HClO_4_ collected during continuous cycling between 0 and 1.2 V. Scan rate: 20 mV s^–1^. Spectra were collected at intervals of 0.6 s (i.e., each spectrum covers an interval of 12 mV), but are shown at intervals of 56 s for the sake of clarity. Resolution: 4.0 cm^–1^. Black lines show the growth of the water bands. Reference spectrum was taken at 0 V.

**Figure 6:**
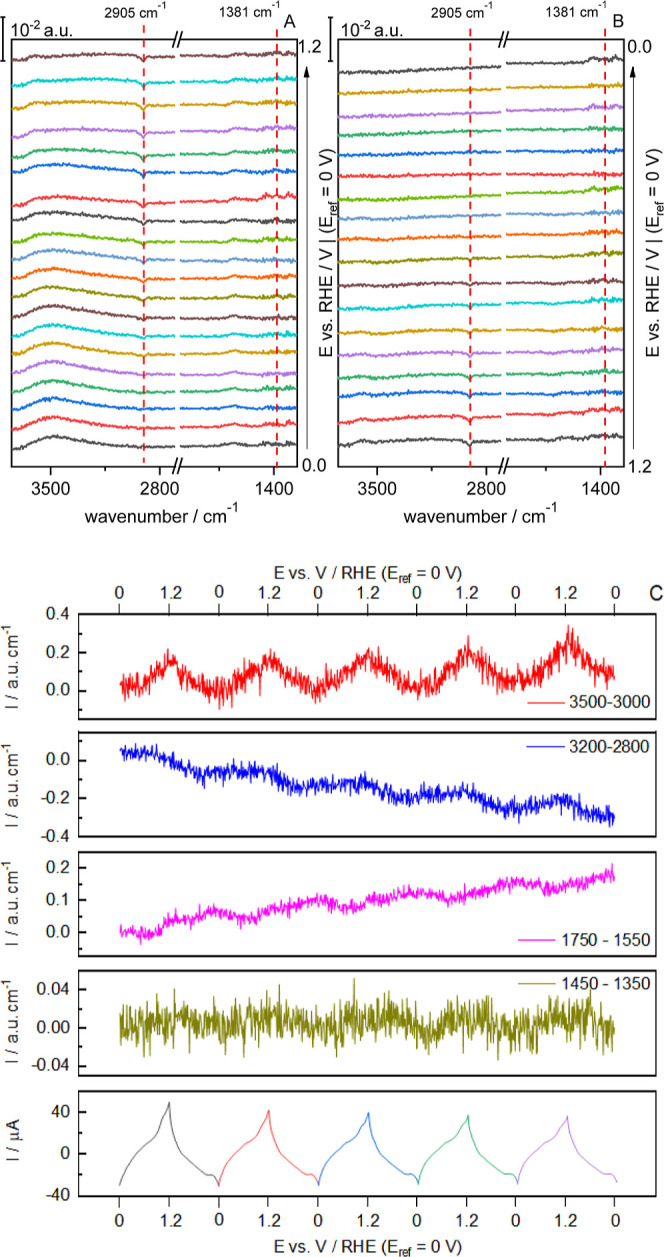
Selected ATR-SEIRA spectra (A,B) of CTAB on Au in 0.1 M HClO_4_ during a cycle voltammogram between 0 and 1.2 V. (A) shows the positive and (B) the negative sweep. Scan rate: 20 mV s^–1^. (C) Dependence of the integrated intensity of selected bands in the ATR-SEIRA spectra of CTAB on Au on the applied potential. Spectra were collected at intervals of 0.6 s (i.e., each spectrum covers an interval of 12 mV). Resolution: 4.0 cm^–1^. Reference spectrum was taken at 0 V.

With the aim of monitoring in real time the potential-induced changes in the adlayer configuration, a series of spectra was obtained while cycling the electrode between 0 and 1.2 V. Since the major changes took place during the first scan, as reported in Figure 3A, the first scan was discarded, and the spectra were referred to the spectrum taken during the second scan at 0 V. Figure 5 shows the whole series. The main feature in the series is the continuous increase upon cycling of the intensity of the bands corresponding to the ν(OH) and the δ(H–O–H) vibrational modes. This increase can be explained by the release of excess CTAB into the bulk of the solution, bringing more water to the surface. The general trend of the bands 2905 and 1380 cm^–1^ that indicates the reorientation of CTAB cannot be properly distinguished so in Figure 6A,B the second cycle in positive and negative sweep are plotted, respectively. Although the general increase of water bands in only one cycle cannot be detected, an oscillation of these signals with the potential can be observed. Moreover, a negative band at 2905 cm^–1^ starts appearing at ca. 0.5 V in the positive scan direction and its intensity increases with the increasing positive potential. In the negative scan direction, the behaviour is reversed, and the negative band disappears at ca. 0.5 V. On the other hand, and due to the low signal-to-noise ratio, the bands at *ca.* 1380 cm^–1^ are not resolved.

Despite this general trend, there is a superimposed oscillatory behaviour associated with the cycling. This behaviour can be observed in Figure 6C, where the integrated intensities in the regions between 3000 and 3500 and 1550–1750 cm^–1^ are plotted against the applied potential. In each cycle, the intensity of the water bands is larger at the positive potential limit and decreases again when the scan direction is reversed, revealing the detachment of the CTA^+^ layer and the permeation of water through it. The same oscillation can be observed in the region corresponding to the ν(CH) and δ(CH_2_) bands. Although the oscillation representing the signal from the integrations of the ν(CH) bands can be easily associated with the behaviour shown in the spectra, this correlation cannot be properly demonstrated with δ(CH_2_) in these initial cycling stages. Nevertheless, the triangular waves superimposed to the general trend reflects the attachment/detachment process of the layer as the charge of the electrode changes.

**Figure 7:**
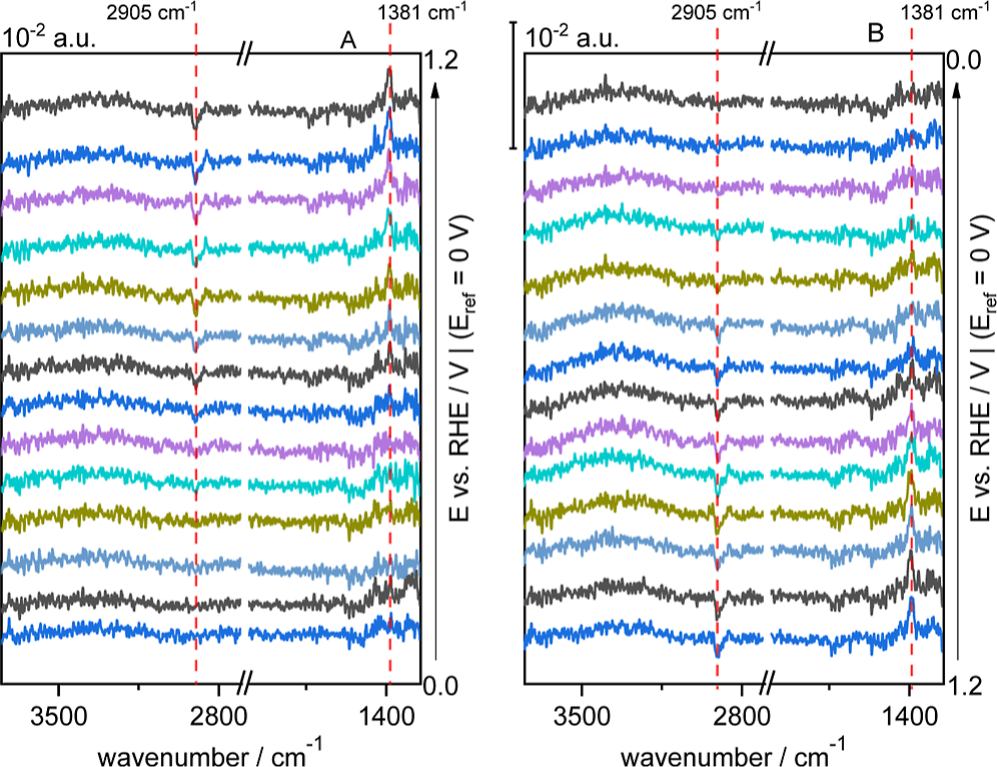
Selected ATR-SEIRA spectra of CTAB on Au in 0.1 M HClO_4_ recorded during a cyclic voltammogram at 10 mV s^–1^ between 0 and 1.2 V in the positive (A) and negative (B) sweep after 100 cycles at 50 mV s^–1^. Spectra were collected at intervals of 0.2 s (i.e., each spectrum spans over 10 mV). Resolution: 4.0 cm^–1^. The reference spectrum was the spectrum at 0 V of this series.

However, the general trend of the ν(CH) and δ(CH_2_) bands is easily seen when cycles are long enough for the adsorption layers to rearrange, as seen in Figure 7 which shows a series of spectra over a complete cycle after 100 cycles. Here, the growth of the δ(CH_2_) bands is seen to follow the same behavior of the ν(CH) bands, but in the opposite direction, a tendency that occurs when CTAB is adsorbed on Au(111).^20^ This suggests that the adlayer reorientated when it detached from the surface and precipitated into CTAClO_4_.

### 3.5 Potentiostatic SERS Experiments

**Figure 8:**
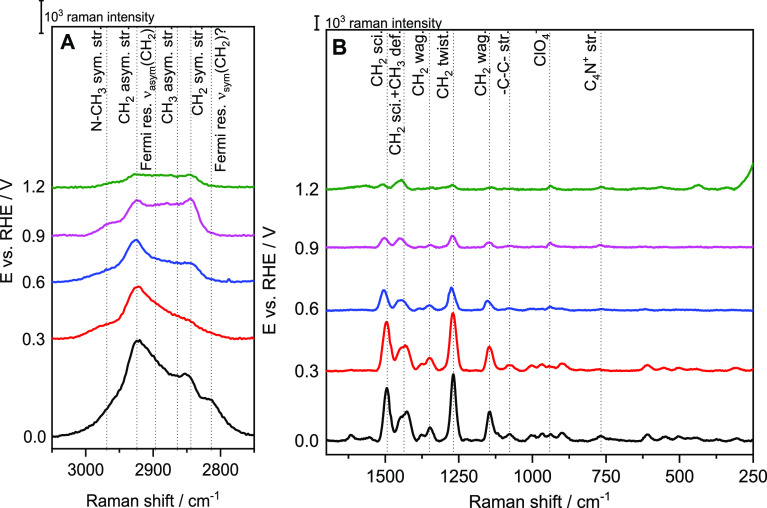
Potential-dependent SER spectra of CTAB-modified Au on the high (A) and mid-low (B) frequency region.

The attachment/detachment of the CTA^+^ layer can also be followed by SERS (Figure 8), because the surface enhancement rapidly declines with the distance.^42^ The selectivity of SERS to the surface species allows to obtain the absolute spectra of the adlayer. Overall, the spectra show a decrease of the Raman intensity with increasing potential in the whole frequency range. The C–H stretching region of CTA^+^ (Figure 8A and Table 2) contains the CH_2_ asymmetric (2925 cm^–1^), the N–CH_3_ symmetric (2968 cm^–1^), the Fermi resonance of the CH_2_ (2895 cm^–1^), the CH_3_ asymmetric (2862 cm^–1^), the CH_2_ symmetric (2843 cm^–1^) and the CH_3_ symmetric stretching (2850 cm^–1^).^43^ At sufficiently negative potential, the bands corresponding to the stretching of the CH_3_ groups appear obscured by the intense peaks of the CH_2_ stretching bands. The more positive the potential, the better the bands are resolved for the CH_3_ groups, i.e., the ratio between the intensity of the CH_3_/CH_2_ bands increases. The bands corresponding to these two modes have approximately the same intensity at 1.2 V, suggesting a reorientation of the adlayer. A clear decrease of the Raman intensities with increasing positive potential is also observed in the mid-low wavenumber region (Figure 5B). In this region, CH_2_ bending modes between 1550 and 1050 cm^–1^,^44−46^ the −C–C– stretching region at 1160–1040 cm^–1^,^43,46−48^ and the N–CH_3_ stretching of the head group at 800–740 cm^–1^ can be observed.^19,43,49^ Finally, the N–CH_3_ stretching mode intensity is the least affected by the potential. All these intensity changes are clear indication of the progressive detachment of CTA^+^ from the surface.

## 4 Conclusions

We have used a combination of spectroelectrochemical techniques to study the behaviour of CTAB adsorbed on gold electrodes in perchloric acid solutions. The desorption of the bromide ions in the negative limit and their oxidation in the positive limit of the voltammograms during cycling results in their exchange with ClO_4_^–^, which form ionic pairs with CTA^+^. This results in a continued rearrangement of the CTA^+^ adlayer that leads to the formation of micro-dominions of different types of hydrogen-bonded water populations throughout the adlayer. After prolonged cycling, the adlayer stabilises enough to allow water molecules to permeate freely toward the gold surface at positive potentials, due to the repulsion experienced by the CTA^+^ layer by the positive charge on the electrode surface. SERS and ATR-SEIRAS consistently reveal the detachment and reorientation of the adsorbed CTA^+^ layer at positive potentials.
